# Diagnostic accuracy of imaging modalities for primary small-bowel tumors: a systematic review and diagnostic test accuracy meta-analysis

**DOI:** 10.3389/fonc.2026.1842330

**Published:** 2026-06-26

**Authors:** Qi Xia, Qinghai Li, Chao Ding, Jun Wang

**Affiliations:** 1 Department of General Surgery II, The First People’s Hospital of Daishan County, Daishan, Zhejiang, China; 2 Department of Radiology, The Second Affiliated Hospital, Zhejiang University School of Medicine, Hangzhou, Zhejiang, China; 3 Department of Hepatopancreatobiliary Surgery, The Second Affiliated Hospital, Zhejiang University School of Medicine, Hangzhou, Zhejiang, China; 4 Department of Gastroenterology Surgery, The Second Affiliated Hospital, Zhejiang University School of Medicine, Hangzhou, Zhejiang, China

**Keywords:** diagnostic imaging, diagnostic test accuracy, enterography, meta-analysis, small bowel neoplasms

## Abstract

**Background:**

Primary small-bowel tumors are uncommon and frequently present with non-specific symptoms. Timely and accurate diagnosis relies on a range of radiologic and endoscopy-based modalities, yet their comparative diagnostic performance remains uncertain.

**Methods:**

We performed a systematic review and diagnostic test accuracy meta-analysis. PubMed, Embase, CNKI, and Wanfang databases were searched from inception to August 31, 2025, and the reference lists of included studies and relevant reviews were hand-searched. Two reviewers independently screened studies, extracted data, and assessed the risk of bias using the QUADAS-2 tool. To avoid unit-of-analysis errors, the primary analysis used a de-duplicated dataset with one representative arm per study cohort according to a prespecified hierarchy: the primary or original/consensus reading reported by the source article, then the arm with the largest analyzable 2×2 denominator, and finally the estimate closest to the within-study median diagnostic odds ratio if ties remained. Pooled sensitivity and specificity were estimated using hierarchical bivariate random-effects models, while alternative arms were retained for sensitivity analyses.

**Results:**

Twenty-one studies met the eligibility criteria and were included in the quantitative synthesis. The overall pooled sensitivity was 0.91 (95% CI: 0.87-0.93) and specificity was 0.88 (95% CI: 0.78-0.94), with an area under the summary receiver operating characteristic curve of 0.95 (95% CI: 0.93-0.97). Between-study heterogeneity was substantial. In modality-stratified analyses, dedicated enterography techniques (magnetic resonance enterography and computed tomography enterography) demonstrated the highest comprehensive diagnostic accuracy, outperforming conventional contrast-enhanced CT and functional imaging.

**Conclusions:**

Contemporary imaging modalities exhibit high overall diagnostic performance for primary small-bowel tumors. Enterography-based cross-sectional imaging yielded the most favorable point estimates, although confidence intervals overlapped with those of conventional CT. These findings support CTE and MRE as strong diagnostic options within a multimodality pathway rather than proving clear statistical superiority.

**Systematic review registration:**

https://www.crd.york.ac.uk/PROSPERO/, identifier CRD420251144585.

## Introduction

Primary tumors of the small intestine represent a diagnostically challenging spectrum of diseases. Although the small intestine constitutes most of the gastrointestinal tract, primary small-intestine malignancies are uncommon compared with gastric and colorectal cancer and are frequently diagnosed at an advanced stage (1, 2). In the United States, the SEER program reports an age-adjusted incidence rate of 2.6 per 100,000 persons per year (2018 to 2022) and an age-adjusted mortality rate of 0.4 per 100,000 (2019 to 2023) (3). Contemporary population estimates similarly underscore the clinical burden despite rarity; the American Cancer Society projected 13,920 new diagnoses and 2,060 deaths from small intestine cancer in 2025 (4).

The clinical presentation of this condition is often non-specific, ranging from chronic abdominal pain, anemia or occult bleeding to intermittent obstruction, delaying diagnostic time (1, 2). Moreover, “small-bowel tumor” is not a single entity but a heterogeneous group that includes adenocarcinoma, neuroendocrine tumors, lymphoma and gastrointestinal stromal tumors, each with distinct growth patterns, biology and staging implications (1, 2). Guideline-based management therefore depends on reliable pre-treatment localization, assessment of depth and extraluminal extension, and detection of nodal or distant disease (2).

The diagnostic work-up is constrained by anatomy: conventional upper and lower endoscopy does not fully visualize the small bowel, and symptoms may precede overt complications. Small-bowel capsule endoscopy and device-assisted enteroscopy provide direct mucosal evaluation and are recommended in selected clinical scenarios (for example, suspected small-bowel bleeding), but their availability and diagnostic yield can be influenced by lesion location, stenosis, and the need for staging beyond the lumen (5). Cross-sectional imaging therefore remains central to both detection and staging. Standard abdominopelvic computed tomography (CT) may miss subtle intraluminal or early mural lesions, whereas dedicated enterography techniques (CT enterography and magnetic resonance (MR) enterography) improve luminal distension and optimize mural enhancement, enabling a more comprehensive assessment of the bowel wall and mesentery (6, 7).

Despite significant advancements in imaging technology, reports on the diagnostic accuracy of radiological techniques for primary small-bowel tumors remain inconsistent (8). This variability largely stems from limitations such as small sample sizes, heterogeneous patient populations, and disparate study protocols, thresholds, and reference standards (9). Consequently, to guide clinicians in selecting optimal imaging strategies and assist radiologists in interpretation (5), there is a critical need for quantitative, modality-specific estimates of sensitivity and specificity that rigorously account for between-study heterogeneity.

We therefore conducted a systematic review and diagnostic test accuracy meta-analysis to synthesize evidence on the performance of contemporary imaging modalities for the diagnosis of primary small-bowel tumors. The study was planned and reported in accordance with the Preferred Reporting Items for Systematic Reviews and Meta-Analyses for Diagnostic Test Accuracy (PRISMA-DTA) statement. Methodological quality was appraised using the Quality Assessment of Diagnostic Accuracy Studies-2 (QUADAS-2) tool, and pooled estimates were derived using bivariate hierarchical models to account for the interdependence of sensitivity and specificity (10–14).

## Methods

This systematic review and diagnostic test accuracy meta-analysis followed the PRISMA-DTA statement (10) and its explanation/elaboration documents (**Supplementary Table 1**).

Search strategy: PubMed, Embase, CNKI and Wanfang were searched from inception to August 31 2025. We combined controlled vocabulary and free-text terms for small-bowel tumors/neoplasms with terms for diagnostic modalities (e.g., CT, CT enterography, MRI/MR enterography, PET/CT, capsule endoscopy, enteroscopy). No language restriction was applied during screening. To minimize retrieval bias, we additionally hand-searched the reference lists of all included studies and relevant reviews. The full search strategies for each database are provided in **Supplementary Methods**.

Eligibility criteria: We included studies enrolling adults with suspected or confirmed primary small-bowel tumors who underwent an index diagnostic modality, with a reference standard based on histopathology (surgical or endoscopic) and/or a composite reference standard incorporating clinical follow-up. Studies had to report (or allow reconstruction of) 2×2 contingency tables (True Positive (TP), False Positive (FP), False Negative (FN), True Negative (TN)). Case reports, reviews, and studies without extractable DTA data were excluded.

Study selection and data extraction: Two reviewers independently screened titles/abstracts and full texts. Using a standardized form, we extracted study characteristics (design, setting, tumor spectrum, index test protocol, main radiological findings when reported, reference standard) and 2×2 data for each eligible test arm. For studies reporting multiple arms within the same cohort, all arms were extracted, but only one representative arm was retained in the primary de-duplicated dataset according to a prespecified hierarchy: (i) the primary index test or original/consensus reading identified by the source article; (ii) if no primary arm was stated, the arm with the largest analyzable 2×2 denominator; and (iii) if ties persisted, the arm whose diagnostic odds ratio was closest to the within-study median, rather than the most optimistic result. Disagreements were resolved by consensus or a third reviewer.

Risk of bias and applicability: Risk of bias was assessed using QUADAS-2 across four domains (patient selection, index test, reference standard, flow and timing) (12). Applicability concerns were judged for the first three domains.

Statistical analysis: Primary pooling used a hierarchical bivariate random-effects model to jointly summarize sensitivity and specificity and to derive the summary receiver operating characteristic (SROC) curve. To prevent unit-of-analysis errors and the artificial inflation of statistical weights from overlapping data, the primary analysis utilized the de-duplicated dataset described above, whereas all alternative or intersecting arms were preserved for sensitivity analyses. Zero cells were handled at the model level when required, rather than by altering the raw 2×2 counts. Positive and negative likelihood ratios (LR+ and LR-), diagnostic odds ratio (DOR), and post-test probabilities were derived from pooled estimates. Heterogeneity was assessed with bivariate I² statistics for sensitivity and specificity and by evaluating threshold effects. We explored heterogeneity with pre-specified subgroup analyses and meta-regression (e.g., study design and sample size). Small-study effects were assessed using Deeks’ funnel plot asymmetry test.

To rigorously evaluate the robustness of our primary findings, a sensitivity analysis was subsequently conducted. In this step, dataset restrictions were relaxed to include all available, potentially intersecting test arms (e.g., data evaluating different imaging modalities or involving distinct readers within the same patient cohort) into the bivariate random-effects model. Analyses were performed in Stata using the user-written commands MIDAS (15) and metadta (16).

## Results

### Search results

**Figure 1:**
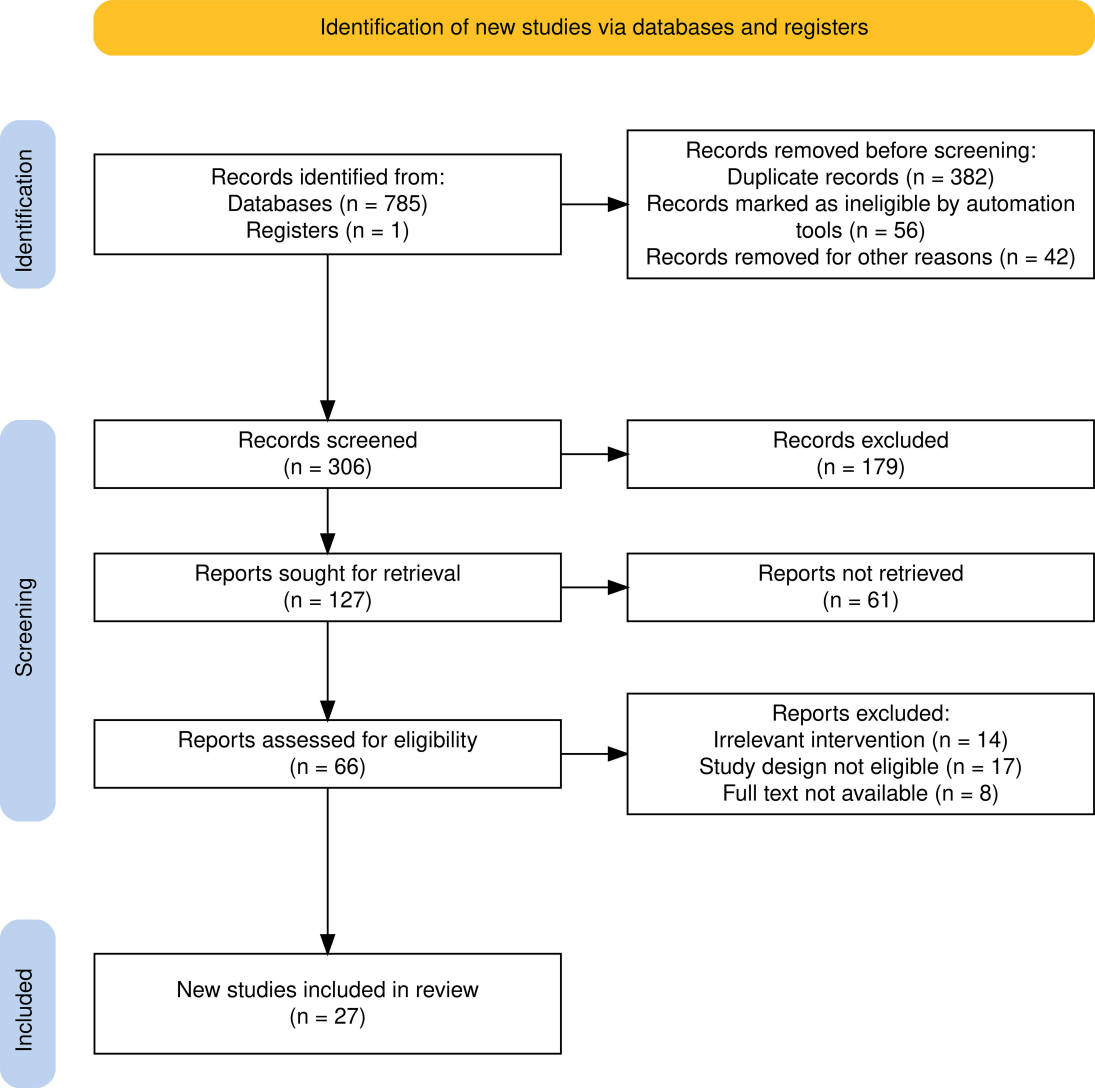
PRISMA flow diagram.

The study selection process is illustrated in **Figure 1**. A total of 786 records were initially identified from databases (n=785) and registries (n=1). Following the removal of 382 duplicates, 404 records were screened based on titles and abstracts. Of these, 338 were excluded, leaving 66 full-text articles for eligibility assessment. Ultimately, 21 studies (17–37) meeting the inclusion criteria were included in the systematic review, contributing 38 test arms to the quantitative synthesis.

### Baseline characteristics of included studies

The detailed characteristics of the 21 included studies are summarized in **Table 1**. Geographically, the studies were predominantly conducted in China, with additional contributions from the USA, Italy, Egypt, and South Korea, reflecting a degree of global diversity. Regarding study design, the majority were retrospective analyses, while a smaller subset employed a prospective design.

**Table 1:** Baseline characteristics of the included studies.

Study	Country	Study design	Number of patients	Imaging method	Number of DTA samples	TP	FP	FN	TN	Number of positive cases
Alaa Mohamed Reda2023	Egypt	PS	69	MRE	69	47	2	4	16	51
Alaa Mohamed Reda2023	Egypt	PS	69	MRE	69	51	1	0	17	51
Alessandro Bonomi2024	Italy	RS	56	CTE	56	48	4	2	2	50
Alessandro Bonomi2024	Italy	RS	56	^68^Ga DOTATATE PETCT	56	47	3	3	3	50
Changhui Xie2012	China	RS	44	^18^F FDG PETCT	44	24	2	1	17	25
Changhui Xie2012	China	RS	44	^18^F FDG PETCT	44	24	1	1	18	25
Chengli Meng2021	China	RS	140	MRE	139	49	0	1	89	50
Chengli Meng2021	China	RS	140	CTE	133	46	0	4	83	50
Chi Zhang2023	USA	RS	104	^68^Ga DOTATATE PETCT	104	24	4	29	47	53
Chi Zhang2023	USA	RS	104	^68^Ga DOTATATE PETCT	104	38	12	15	39	53
Chongyang Ding2015	China	RS	47	^8^F FDG PETCT	47	38	1	3	5	41
Decou Fu2024	China	RS	60	Exray	60	33	9	9	9	42
Decou Fu2024	China	RS	60	CTE	60	39	2	3	16	42
Derek Tang2024	USA	PS	174	SBCE	145	28	3	4	110	32
Derek Tang2024	USA	PS	174	^18^F DOPA PETCT	142	27	0	5	110	32
Enzhao Tian2018	China	RS	46	CT	46	6	8	1	31	7
Hao Feng2022	China	RS	70	MRE	70	48	1	2	19	50
Hao Feng2022	China	RS	70	CTE	70	49	3	1	17	50
Hongrou Ye2023	China	RS	88	DBE	88	51	1	6	30	57
Hongrou Ye2023	China	RS	88	CT	88	51	3	6	28	57
Ji Eun Lee2024	Korea	RS	90	CT+MRI	90	33	4	3	50	36
Ji Eun Lee2024	Korea	RS	90	CT+MRI	90	32	3	4	51	36
Ji Eun Lee2024	Korea	RS	90	CT+MRI	90	31	4	5	50	36
Ji Eun Lee2024	Korea	RS	90	CT	90	24	6	12	48	36
Ji Eun Lee2024	Korea	RS	90	CT	90	25	8	11	46	36
Ji Eun Lee2024	Korea	RS	90	CT	90	20	7	16	47	36
Jiajia Hu2007	China	RS	112	^8^F FDG PETCT	21	16	0	0	5	18
Jiajia Hu2012	China	RS	22	^8^F FDG PETCT	22	16	1	1	4	17
Na Feng2023	China	RS	98	CT	76	20	12	2	42	22
Na Feng2023	China	RS	98	CT	44	19	3	0	22	19
Peixue Wang2023	China	RS	42	DBE	42	32	3	6	1	38
Peixue Wang2023	China	RS	42	CTE+DBE	42	37	4	1	0	38
Peixue Wang2023	China	RS	42	CTE	42	32	2	6	2	38
Tao Zhou2015	China	RS	53	CT	53	27	3	2	21	29
Tiannv Lee2023	China	RS	47	^8^F FDG PETCT	47	37	3	4	3	41
Won Shik Kim2023	Korea	RS	13	DBE	13	2	0	0	11	2
Yuanzhi Fu2015	China	RS	57	CT	56	25	2	2	27	27
Yuheng Liu2024	China	RS	42	^18^F FDG PETCT	42	31	0	11	0	42

PS, Prospective Study; RS, Retrospective Study; SBCE, Small Bowel Capsule Endoscopy; MRE, Magnetic Resonance Enterography; Exray, X-ray Imaging; DBE, Double Balloon Enteroscopy; CTE, Computed Tomography Enterography; CT, Computed Tomography; MRI, Magnetic Resonance Imaging; ^68^Ga_DOTATATE PETCT, Gallium-68 DOTATATE Positron Emission Tomography/Computed Tomography; ^18^F_FDG PETCT, Fluorine-18 Fluorodeoxyglucose Positron Emission Tomography/Computed Tomography; ^18^F_DOPA PETCT, Fluorine-18 Dihydroxyphenylalanine Positron Emission Tomography/Computed Tomography; TP, True Positive; FP, False Positive; FN, False Negative; TN, True Negative; DTA, Diagnostic Test Accuracy.

The meta-analysis encompassed a broad spectrum of contemporary imaging modalities, primarily including CT enterography (CTE), MR enterography (MRE), and double-balloon enteroscopy (DBE). Additionally, several studies evaluated the diagnostic performance of functional imaging (e.g., ^18^F-FDG PET/CT, ^68^Ga-DOTATATE PET/CT, and ^18^F-DOPA PET/CT) and small-bowel capsule endoscopy (SBCE). The pathological spectrum included neuroendocrine tumors, gastrointestinal stromal tumors, lymphoma, ampullary malignancy, peritoneal carcinoma, and mixed or unspecified small-bowel lesions. When descriptive imaging findings were reported, common clues included focal or asymmetric mural thickening, hyperenhancing intraluminal or polypoid masses, luminal narrowing or obstruction, intussusception, exophytic submucosal growth, and mesenteric desmoplastic change in neuroendocrine disease. Sample sizes varied considerably across studies, ranging from 13 to 174 participants, reflecting the rarity of primary small-bowel tumors and variations in case volume across different clinical centers.

### Risk of bias and applicability

**Figure 2:**
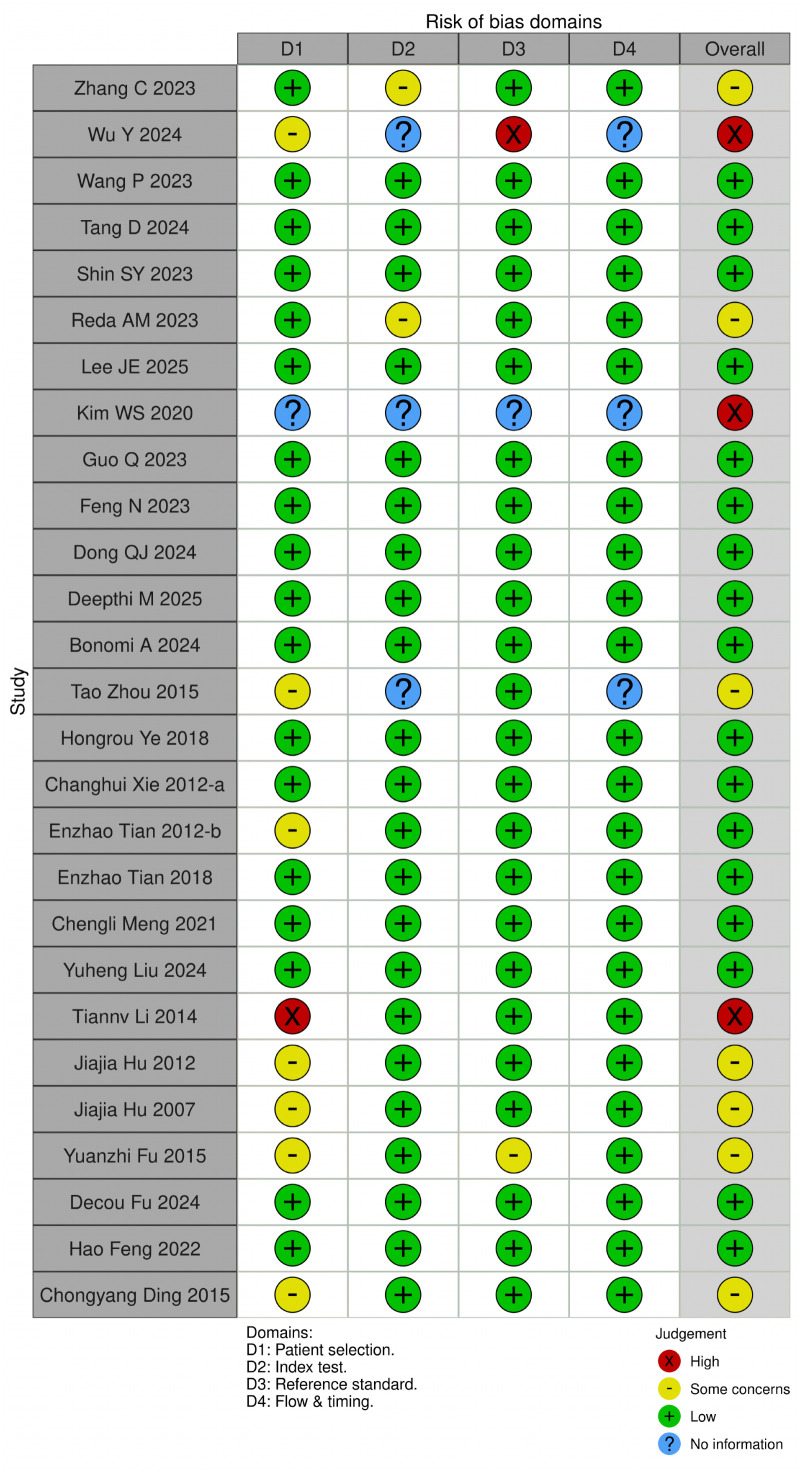
Methodological quality summary. Bar chart presenting the risk of bias and applicability concerns across the 21 included studies. The graph displays the proportion of studies classified as low risk, high risk, or having some concerns for each domain assessed by the QUADAS-2 tool.

Methodological quality was assessed using the QUADAS-2 tool, with domain-level judgments summarized in **Figure 2** and study-specific details provided in **Supplementary Table 2**. Overall, 9 of the 21 included studies (42.9%) were classified as having a low risk of bias across all domains. Eight studies (38.1%) were rated as having some concerns, largely due to incomplete reporting regarding the index test (e.g., blinding or threshold pre-specification). The remaining 4 studies (19.0%) were deemed at high risk of bias, primarily driven by deficiencies in patient selection or reference standard application. Accordingly, the pooled estimates should be interpreted with appropriate caution and in light of study-quality heterogeneity.

### Overall diagnostic accuracy

**Figure 3:**
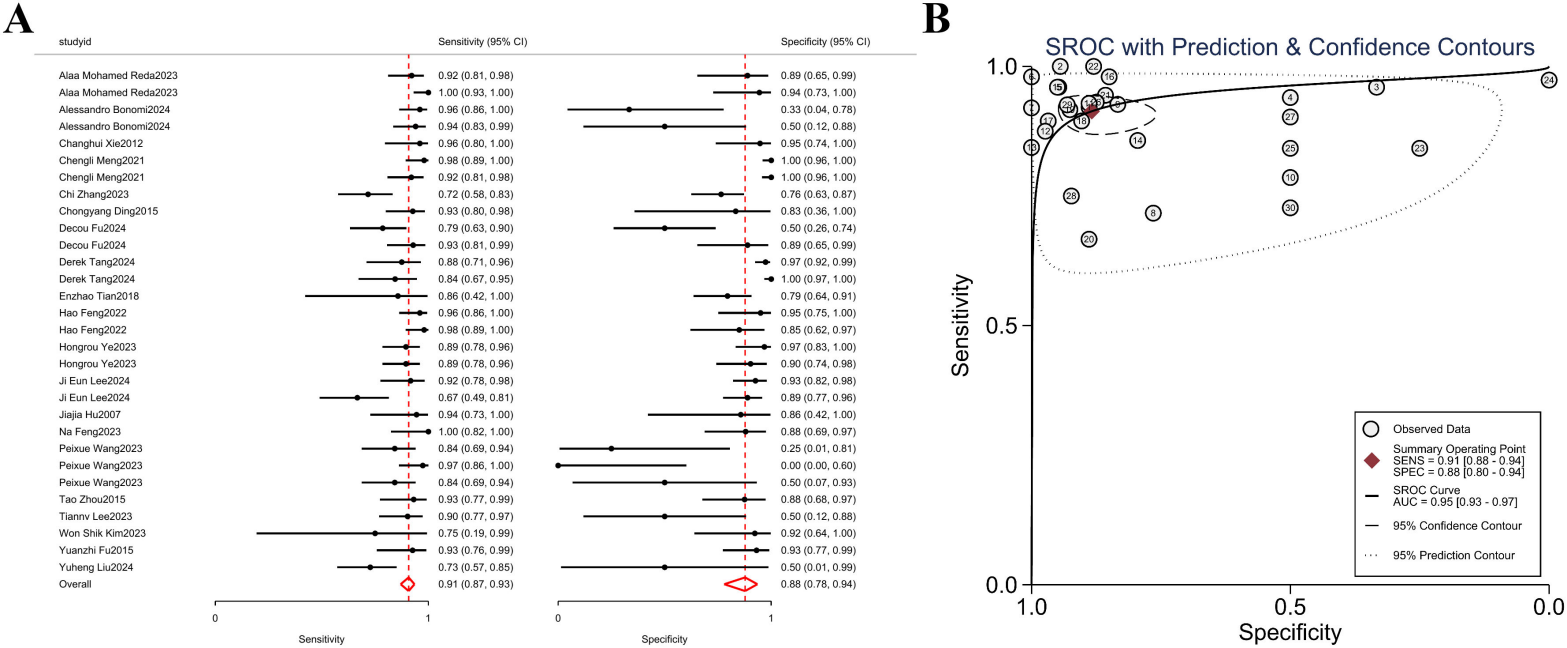
Overall diagnostic accuracy of imaging modalities for primary small-bowel tumors. **(A)** Forest plots of the pooled sensitivity and specificity derived from the bivariate random-effects model. The squares represent individual study estimates with their 95% confidence intervals (CIs), and the diamonds represent the pooled estimates. **(B)** Summary Receiver Operating Characteristic (SROC) curve. The plot shows the summary operating point (solid square) surrounded by the 95% confidence region (dotted line) and the 95% prediction region (dashed line).

The quantitative synthesis included 20 studies (contributing 30 datasets) comprising 1,158 reference-positive and 922 reference-negative examples. Individual dataset estimates varied widely, with sensitivity ranging from 0.67 to 1.00 and specificity from 0.00 to 1.00 (**Table 2**). Based on the bivariate random-effects model, the pooled sensitivity was 0.91 (95% CI: 0.87-0.93) and the pooled specificity was 0.88 (95% CI: 0.78-0.94) (**Figure 3A**). The SROC curve indicated high overall diagnostic accuracy, with an Area Under the Curve (AUC) of 0.95 (95% CI: 0.93-0.97). The summary operating point corresponded to a sensitivity of 0.91 (95% CI: 0.87-0.93) and a specificity of 0.88 (95% CI: 0.78-0.94) (**Figure 3B**).

**Table 2:** Summary diagnostic accuracy estimates (hierarchical models).

Stratum	Sensitivity (95% CI)	Specificity (95% CI)	Model/notes	Source
Overall (primary)	0.91 (0.87–0.93)	0.88 (0.78–0.94)	Bivariate random-effects	MIDAS / metadta
Prospective studies	0.92 (0.87–0.95)	0.98 (0.95–0.99)	Bivariate random-effects	metadta
Retrospective studies	0.91 (0.86–0.94)	0.85 (0.74–0.92)	Bivariate random-effects	metadta
CT enterography (CTE)	0.94 (0.87–0.97)	0.86 (0.28–0.99)	Subgroup analysis	metadta
MR enterography (MRE)	0.96 (0.89–0.98)	0.98 (0.83–1.00)	Subgroup analysis	metadta
Contrast-enhanced CT	0.90 (0.78–0.96)	0.88 (0.82–0.92)	Subgroup analysis	metadta
Capsule endoscopy (SBCE)	0.88 (0.71–0.96)	0.97 (0.92–0.99)	Single study estimate	metadta
Double-balloon enteroscopy (DBE)	0.87 (0.78–0.93)	0.86 (0.36–0.99)	Subgroup analysis	metadta
^18^F-FDG PET/CT	0.91 (0.80–0.96)	0.78 (0.51–0.93)	Subgroup analysis	metadta
^18^F-DOPA PET/CT	0.84 (0.67–0.95)	1.00 (0.97–1.00)	Single study estimate	metadta
^68^Ga-DOTATATE PET/CT	0.83 (0.74–0.89)	0.74 (0.61–0.83)	Subgroup analysis	metadta
Conventional radiography (SBC)	0.79 (0.63–0.90)	0.50 (0.26–0.74)	Single study estimate	metadta
CT+MRI (combined)	0.92 (0.78–0.98)	0.93 (0.82–0.98)	Single study estimate	metadta
CTE+DBE (combined)	0.97 (0.86–1.00)	0.00 (0.00–0.60)	Single study estimate	metadta

CI, confidence interval; CT, computed tomography; CTE, computed tomography enterography; DBE, double-balloon enteroscopy; MRE, magnetic resonance enterography; MRI, magnetic resonance imaging; PET, positron emission tomography; SBC, small bowel contrast radiography; SBCE, small bowel capsule endoscopy.

Overall (primary) accuracy estimates were derived from the de-duplicated dataset to prevent unit-of-analysis errors. Subgroup analyses were performed using the bivariate random-effects model where sufficient data were available. "Single study estimate" denotes absolute measures of diagnostic accuracy extracted from an individual test arm where quantitative synthesis (meta-analysis) was not applicable due to the limited number of studies. MIDAS and metadta are user-written commands in Stata utilized for the diagnostic test accuracy meta-analysis.

### Threshold effect and influence diagnostics

**Figure 4:**
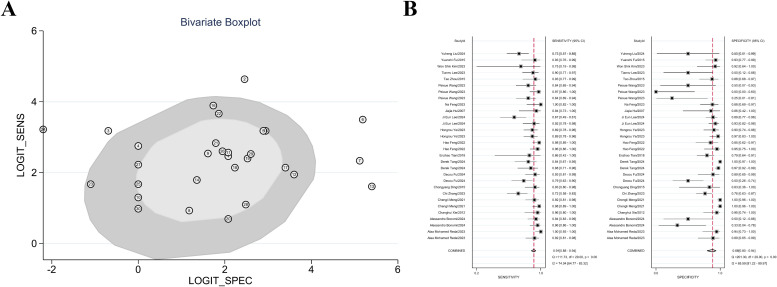
Threshold effect and heterogeneity analysis. **(A)** Bivariate boxplot of logit-transformed sensitivity versus specificity. The linear arrangement of studies suggests a negative correlation between indices, indicating a threshold effect. The inner and outer ellipses represent the 95% confidence region and prediction region, respectively. **(B)** Study-level Forest plots highlighting the substantial between-study heterogeneity and the dispersion of accuracy estimates across included datasets.

Evaluation of the threshold effect revealed a positive correlation between logit-transformed sensitivity and specificity (Spearman correlation coefficient = 0.23). The proportion of heterogeneity likely due to the threshold effect was calculated to be only 0.05, suggesting that the threshold effect was not the primary driver of inter-study heterogeneity (**Figure 4A**). Influence analysis identified a small number of studies outside the 95% concentration ellipse. Notably, one outlier dataset reported a specificity of 0.00 (95% CI: 0.00-0.60), reflecting a high false-positive rate that disproportionately affected the pooled specificity estimate. In sensitivity analyses excluding datasets with specificity <0.50, the pooled specificity improved, while sensitivity remained stable.

### Heterogeneity analysis

Statistically significant between-study heterogeneity was observed (Cochran’s Q = 39.66, P < 0.001), with an overall I² of 95% (95% CI: 91%-99%) (**Figure 4B**). Study-level visualizations highlighted the dispersion of accuracy estimates, contrasting series with low sensitivity or high false-positive rates against those utilizing dedicated enterographic techniques, which consistently achieved high sensitivity and specificity.

### Subgroup analyses by country

**Figure 5:**
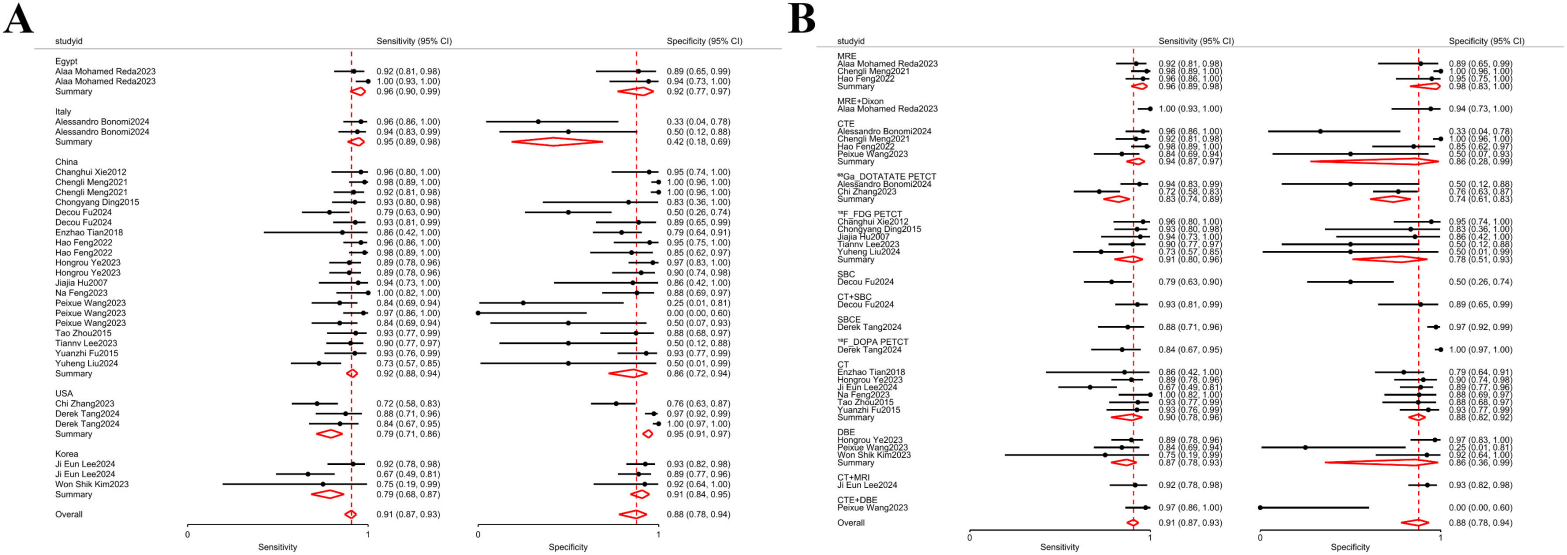
Subgroup analyses stratified by geographic location and imaging modality. **(A)** Forest plots of diagnostic accuracy stratified by country (e.g., China, USA, Korea, etc.), showing pooled estimates for the Chinese subgroup and individual estimates for other regions. **(B)** Forest plots stratified by imaging modality, comparing the diagnostic performance of CT enterography (CTE), MR enterography (MRE), conventional CT, functional imaging, and combined strategies.

In analyses stratified by country (**Figure 5A**), China contributed the largest number of datasets and displayed a summary sensitivity of 0.92 (95% CI: 0.88-0.94) and specificity of 0.86 (95% CI: 0.72-0.94), consistent with the overall pooled estimates. Within-country heterogeneity remained evident (**Supplementary Figure 1A**). For instance, while some series achieved high accuracy (e.g., sensitivity of 1.00), others reported significantly lower specificity (e.g., 0.00 or 0.25), suggesting that differences in technique, threshold definition, and patient spectrum characteristics may contribute to variability beyond geographic factors alone.

### Subgroup analyses by imaging modality

Modality-stratified analyses were summarized in **Figure 5B**. MRE showed the highest point estimates, with a summary sensitivity of 0.96 (95% CI: 0.89-0.98) and specificity of 0.98 (95% CI: 0.83-1.00). CTE yielded a summary sensitivity of 0.94 (95% CI: 0.87-0.97), but its specificity estimate was imprecise at 0.86 (95% CI: 0.28-0.99). Conventional contrast-enhanced CT demonstrated a sensitivity of 0.90 (95% CI: 0.78-0.96) and specificity of 0.88 (95% CI: 0.82-0.92). Functional imaging showed divergent results: ^68^Ga-DOTATATE PET/CT showed a pooled sensitivity of 0.83 (95% CI: 0.74-0.89) and specificity of 0.74 (95% CI: 0.61-0.83), while ^18^F-DOPA PET/CT (one dataset) achieved a specificity of 1.00 and was therefore interpreted descriptively. For the combined CTE plus DBE arm, the observed specificity of 0.00 arose from a very small non-tumor subgroup (TN = 0, FP = 4) rather than from a missing control group; this arm was retained because a reconstructable 2×2 table was available, but the estimate is unstable and should not be overinterpreted.

### Subgroup analyses by PET tracer

**Figure 6:**
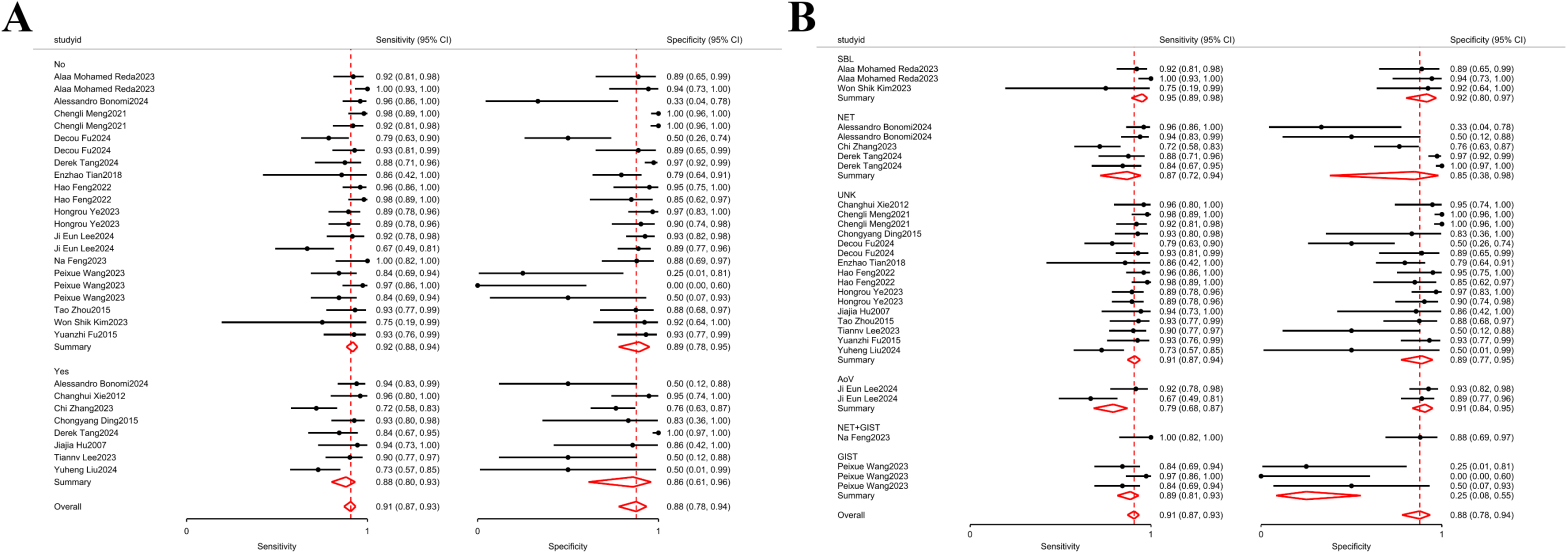
Subgroup analyses stratified by the use of PET tracers and tumor type. **(A)** Forest plots comparing the diagnostic accuracy between conventional anatomical/structural imaging modalities (No PET tracer) and functional imaging modalities utilizing PET tracers (Yes). **(B)** Forest plots of diagnostic accuracy categorized by specific tumor histologies or classifications, including generic small bowel lesions (SBL), neuroendocrine tumors (NET), gastrointestinal stromal tumors (GIST), ampulla of Vater tumors (AoV), and unknown/mixed types (UNK). Squares represent individual dataset estimates with their 95% confidence intervals (CIs), and diamonds represent the pooled summary estimates.

To evaluate the impact of functional imaging, studies were stratified by the use of PET tracers (**Figure 6A**). The subgroup comprising conventional anatomical and structural imaging modalities (without PET tracers) demonstrated a robust pooled sensitivity of 0.92 (95% CI: 0.88-0.94) and specificity of 0.89 (95% CI: 0.78-0.95). In comparison, the subgroup utilizing PET tracers (functional imaging, such as ^18^F-FDG or ^68^Ga-DOTATATE) yielded a slightly lower pooled sensitivity of 0.88 (95% CI: 0.80-0.93) and specificity of 0.86 (95% CI: 0.61-0.96). This suggests that while functional imaging is valuable for specific metabolic evaluations, contemporary structural imaging maintains a highly favorable and stable overall diagnostic profile for primary detection.

### Subgroup analyses by tumor type

Diagnostic performance stratified by histological classification is presented in **Figure 6B**. Datasets evaluating generic small bowel lesions (SBL) and mixed/unknown types (UNK) achieved excellent diagnostic accuracy (SBL: sensitivity 0.95, specificity 0.92; UNK: sensitivity 0.91, specificity 0.89). For specific histologies, the neuroendocrine tumor (NET) subgroup yielded a pooled sensitivity of 0.87 (95% CI: 0.72-0.94) and specificity of 0.85 (95% CI: 0.38-0.98). Tumors located at the ampulla of Vater (AoV) exhibited a lower sensitivity of 0.79 (95% CI: 0.68-0.87) but maintained a high specificity of 0.91 (95% CI: 0.84-0.95). Notably, while the gastrointestinal stromal tumor (GIST) subgroup maintained high sensitivity (0.89), its pooled specificity was remarkably low (0.25; 95% CI: 0.08-0.55), indicating a significantly elevated risk of false positives, which may be driven by the overlapping imaging features of certain benign submucosal lesions or specific threshold definitions in the included cohorts.

### Clinical utility

**Figure 7:**
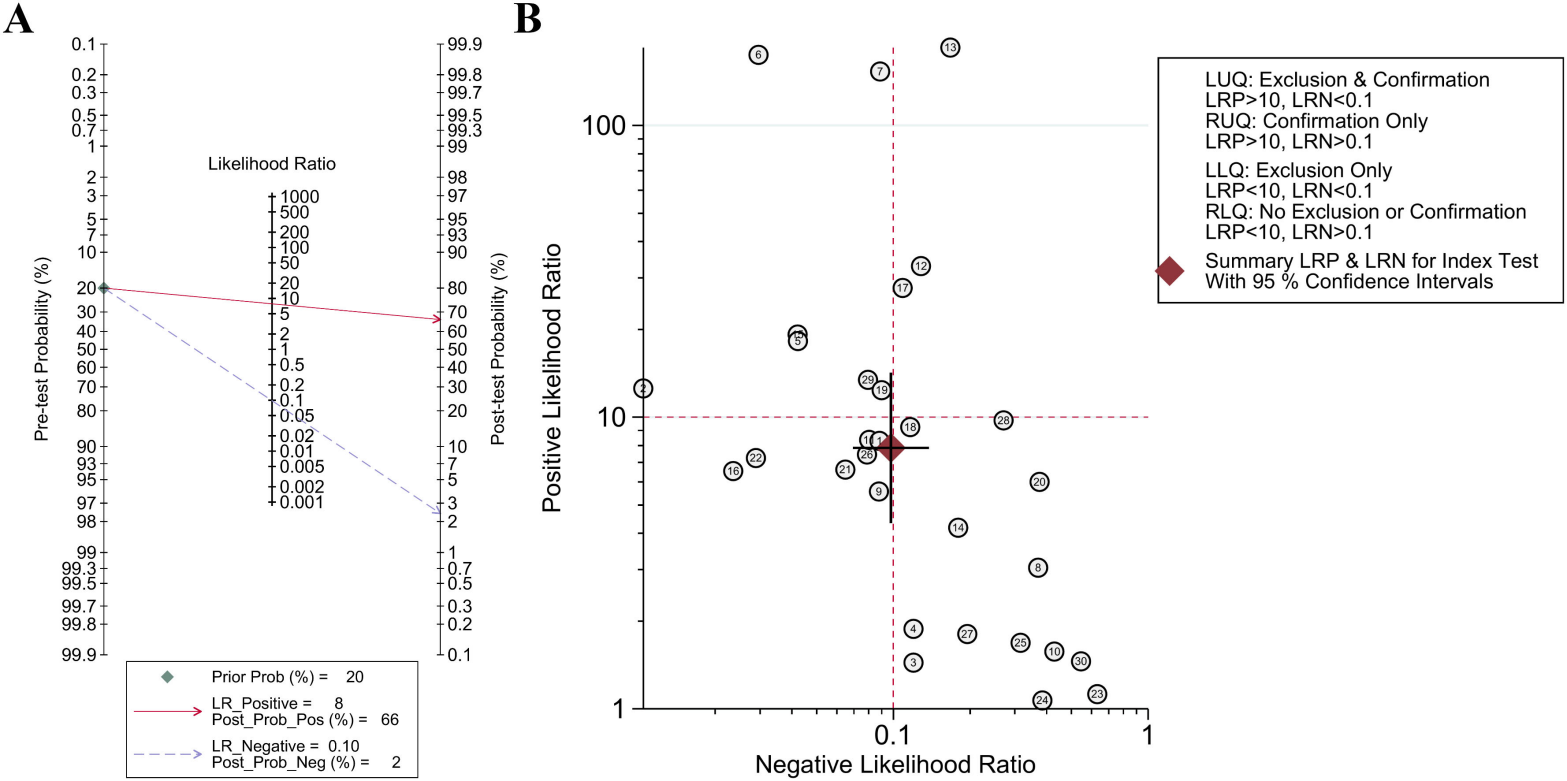
Assessment of clinical utility. **(A)** Fagan’s nomogram for evaluating post-test probabilities. A pre-test probability of 20% was used to calculate the post-test probability of disease following a positive or negative (2%) imaging result, based on the pooled likelihood ratios. **(B)** Likelihood ratio scatter matrix. The plot categorizes studies based on their ability to rule in (LRP > 10) and rule out (LRN < 0.1) disease; studies in the upper-left quadrant (shaded area) represent those meeting stringent utility criteria.

Based on pooled likelihood ratios (positive LR = 7.8; negative LR = 0.10), the Fagan nomogram illustrated that for a pre-test probability of 20%, a positive imaging result significantly increased the post-test probability of disease to 66%, whereas a negative result reduced it to 2% (**Figure 7A**). In the likelihood-ratio scatter matrix, datasets meeting the stringent criteria for excellent clinical utility (ruling in disease: LR+ > 10; ruling out disease: LR- < 0.1) predominantly employed dedicated enterography (CTE or MRE) (**Figure 7B**).

### Publication bias and meta-regression

**Figure 8:**
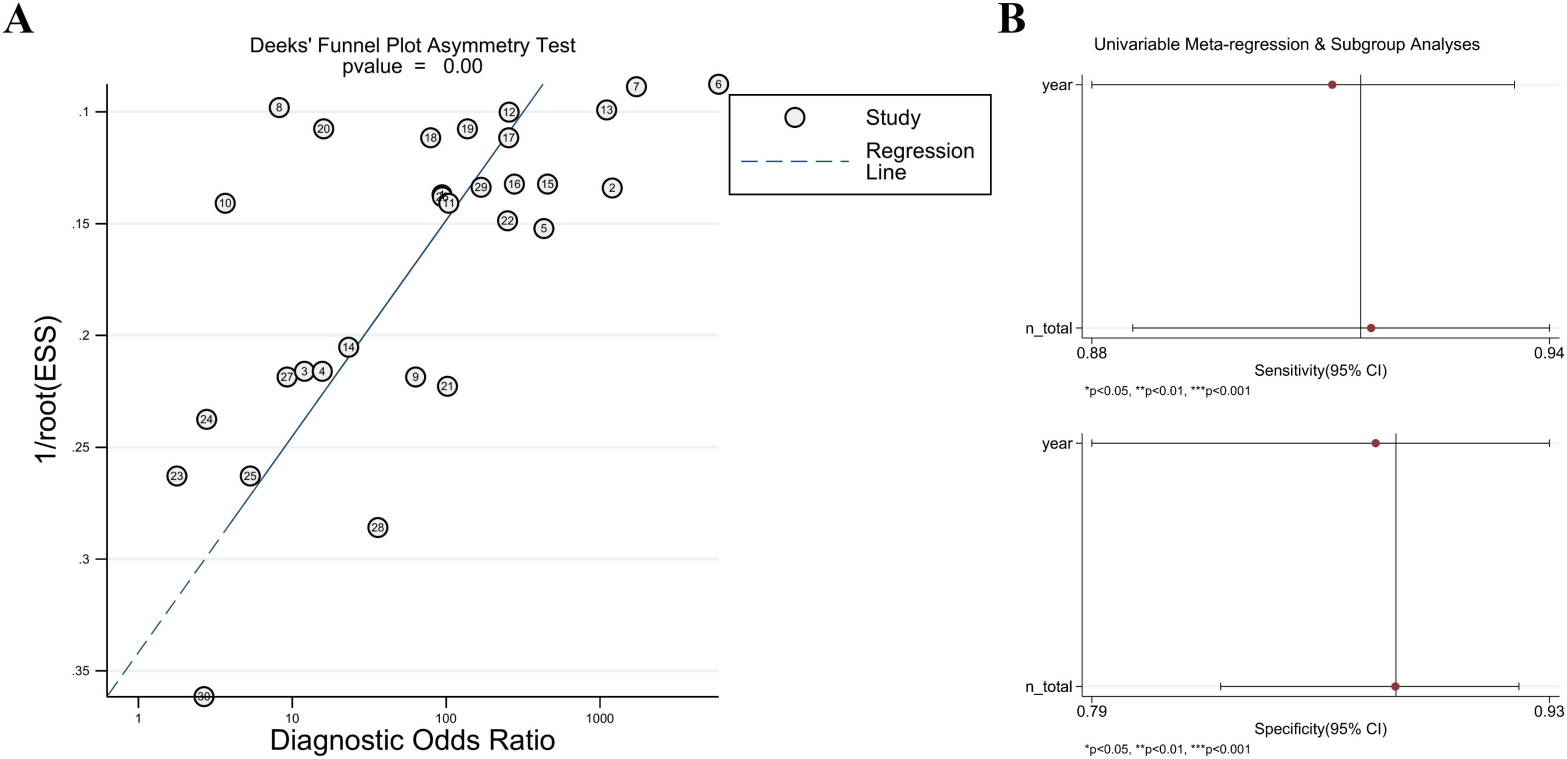
Publication bias and meta-regression analysis. **(A)** Deeks’ funnel plot with superimposed regression line. The asymmetry of the plot (P = 0.03) suggests the potential presence of publication bias or small-study effects. **(B)** Univariable meta-regression plot illustrating the relationship between sample size and specificity. The regression line (slope β = -0.00009) indicates a slight decrease in specificity as study sample size increases (P < 0.05).

The Deeks’ funnel plot asymmetry test yielded a significant bias coefficient (-23.83, P = 0.003), suggesting the potential presence of publication bias or small-study effects (**Figure 8A**). Univariable meta-regression revealed no significant association between publication year and diagnostic accuracy (P = 0.25). However, variations in total sample size were significantly associated with inter-study heterogeneity (P < 0.001), indicating that sample size discrepancies partially explain the variance in diagnostic performance across studies (**Figure 8B**).

### Sensitivity analysis

To evaluate the robustness of the primary analysis results (which were based on the de-duplicated dataset), we performed two complementary sensitivity analyses. First, dataset restrictions were relaxed to include all 38 independent or intersecting test arms extracted from the 21 included studies (without excluding data from different imaging modalities or distinct readers within the same cohort) for a comprehensive evaluation using the bivariate random-effects model. Second, we conducted an additional exclusion analysis in which the four studies judged to be at high risk of bias were removed from the pooled analysis.

### Stability of overall diagnostic performance

Analysis of the complete dataset encompassing all 38 test arms yielded a pooled sensitivity of 0.90 (95% CI: 0.86-0.94) and a pooled specificity of 0.87 (95% CI: 0.78-0.93) (**Supplementary Figure 2**). These results were highly consistent with the primary analysis of the de-duplicated dataset (sensitivity 0.91, specificity 0.88), with minor fluctuations well within their respective 95% confidence intervals. In the additional quality-restricted sensitivity analysis excluding the four high-risk studies, the pooled sensitivity was 0.91 (95% CI: 0.86-0.94) and the pooled specificity was 0.88 (95% CI: 0.78-0.94), indicating that the overall estimates were materially unchanged. Together, these findings suggest that the main conclusions are robust and are not driven solely by overlapping test arms or by the studies judged to be at high risk of bias.

### Consistency of subgroup analysis results

Subgroup analyses based on the full dataset further corroborated the relative relationships of diagnostic performance across various clinical and methodological variables:

Imaging Method: The findings remained consistent with the primary analysis (**Supplementary Figure 3**). Dedicated enterography techniques continued to exhibit the highest comprehensive diagnostic value, with MRE achieving a pooled sensitivity and specificity of 0.96 (95% CI: 0.89-0.98) and 0.98 (95% CI: 0.83-1.00), respectively. CTE yielded a sensitivity of 0.94 (95% CI: 0.87-0.97) and specificity of 0.86 (95% CI: 0.28-0.99). CT had a sensitivity of 0.89 and specificity of 0.85. In terms of functional imaging, ^18^F-FDG PET/CT showed high sensitivity (0.91) but moderate specificity (0.79). Notably, with the inclusion of additional data arms, the sensitivity of ^68^Ga-DOTATATE PET/CT decreased to 0.70 (95% CI: 0.62-0.77) and its specificity to 0.82 (95% CI: 0.74-0.88), suggesting that its sensitivity fluctuates across different subgroups.

Tumor Type: The small bowel lesion (SBL) and mixed/unknown (UNK) subgroups demonstrated high accuracy (SBL: sensitivity 0.95, specificity 0.92; UNK: sensitivity 0.91, specificity 0.88) (**Supplementary Figure 4**). The neuroendocrine tumor (NET) subgroup had a sensitivity of 0.84 and specificity of 0.87. While the gastrointestinal stromal tumor (GIST) subgroup maintained a high sensitivity (0.89), its specificity was extremely low (0.25).

Study Design: Prospective studies continued to significantly outperform retrospective studies in pooled specificity (0.98, 95% CI: 0.95-0.99 vs. 0.85, 95% CI: 0.75-0.92), while their sensitivities were comparable (0.92 vs. 0.90).

PET Tracer: The conventional anatomical/structural imaging groups without PET tracers slightly outperformed those using PET tracers in both sensitivity (0.92 vs. 0.89) and specificity (0.89 vs. 0.86), following the same trend as the primary analysis (**Supplementary Figure 5**).

Country: The Chinese cohort dataset remained predominant, with a sensitivity of 0.92 and specificity of 0.85 (**Supplementary Figure 6**). The USA and Korean cohorts exhibited a “low sensitivity, high specificity” profile (USA: sensitivity 0.69, specificity 0.94; Korea: sensitivity 0.76, specificity 0.90).

Overall, after incorporating all intersecting and repeated test arms, heterogeneity remained substantial (likelihood ratio test p < 0.001), but none of the evaluation metrics underwent fundamental changes compared with the primary analysis. Likewise, exclusion of the four high-risk studies did not materially alter the pooled sensitivity or specificity, supporting the stability of the primary findings.

## Discussion

In this systematic review and DTA meta-analysis of 21 studies comprising approximately 1,474 participants, hierarchical modeling demonstrated robust overall discriminative performance for contemporary imaging in the diagnosis of primary small-bowel tumors. The pooled sensitivity of 0.91 and specificity of 0.88 suggest that modern radiological techniques provide effective separation between neoplastic and non-neoplastic pathologies. From a clinical perspective, the pooled likelihood ratios (positive LR ~8.0; negative LR ~0.1) indicate that a positive imaging finding meaningfully increases post-test probability in patients with moderate pre-test suspicion, whereas a negative result substantially lowers this probability. These operating characteristics support the utility of imaging for potentially de-escalating to alternative diagnostic pathways or surveillance, depending on the specific clinical context.

These findings align with current diagnostic paradigms in which cross-sectional imaging serves as a cornerstone of the work-up for suspected small-bowel neoplasia, particularly when symptoms persist following negative bidirectional endoscopy (oesophagogastroduodenoscopy and colonoscopy). Beyond mere detection, imaging is expected to provide actionable anatomical information, including lesion localization, extraluminal extension, mesenteric involvement, and distant metastases. This directly informs the selection of subsequent modalities, such as capsule endoscopy or device-assisted enteroscopy, as well as surgical planning and oncologic staging (2, 5).

A clinically important implication of our findings is that dedicated enterography techniques yielded numerically higher and more consistent diagnostic estimates than conventional imaging protocols. Both MRE and CTE achieved high sensitivity, but the confidence intervals, particularly for CTE specificity, were wide and overlapped with those of conventional CT. Accordingly, our results support enterography as a strong diagnostic option rather than proving definitive statistical superiority. From a radiological perspective, the included studies most often described tumors as focal or asymmetric mural thickening, hyperenhancing intraluminal masses, polypoid lesions, luminal narrowing or upstream dilatation, intussusception, or exophytic submucosal masses; in neuroendocrine disease, mesenteric desmoplastic reaction and multifocal hyperenhancing ileal lesions were recurring clues. These features help explain why optimized luminal distension and dedicated bowel protocols may improve lesion conspicuity (6, 7, 38).

Functional nuclear imaging modalities represent a distinct diagnostic category with unique operating characteristics. As observed in our heterogeneity analysis, these techniques exhibited variable performance: ^18^F-FDG PET/CT demonstrated high sensitivity (0.91) but moderate specificity (0.78), while ^68^Ga-DOTATATE PET/CT showed lower pooled sensitivity (0.83) and specificity (0.74). This variability likely reflects differences in tracer uptake physiology and target tumor biology. These modalities are most clinically relevant for specific phenotypes, particularly well-differentiated neuroendocrine tumors, where their primary utility lies in staging, detecting occult primaries, and mapping multifocal disease rather than serving as a universal screening tool for all small-bowel masses (9, 39, 40). Consequently, modality selection should remain phenotype-driven and embedded within multidisciplinary algorithms.

Several methodological factors influence the interpretation of these pooled estimates. In the bivariate model, the proportion of heterogeneity likely due to the threshold effect was only 0.05 (with a positive correlation coefficient of 0.23), suggesting that non-threshold factors, such as variations in patient spectrum, scanner generation, protocol design, and reader expertise, were the main contributors. Our QUADAS-2 assessment also showed that 4 of 21 studies (19.0%) were at high risk of bias and another 8 (38.1%) had some concerns, most often because of patient-selection issues, incompletely prespecified index-test thresholds, or reference-standard limitations. These study-quality concerns reduce certainty in the pooled estimates and argue against over-interpreting small numerical differences between modalities. At the same time, the additional sensitivity analysis excluding all four high-risk studies yielded pooled estimates that were essentially unchanged (sensitivity 0.91, specificity 0.88), which provides reassurance that the main conclusions are not driven solely by the lower-quality studies.

This meta-analysis has specific limitations that warrant consideration. First, a substantial proportion of the included studies originated from a single geographic region (China), which may limit the generalizability of the findings to Western populations with potentially different body mass index distributions or disease prevalence profiles. Second, the evidence base comprised heterogeneous tumor types (e.g., small bowel adenocarcinoma, neuroendocrine tumors, GISTs, lymphoma, ampullary lesions) and mixed clinical indications, which may dilute modality-specific performance for any single histology. Third, despite the use of the bivariate hierarchical model to account for the correlation between sensitivity and specificity, residual heterogeneity remained high, likely due to unmeasured differences in scanner generation, radiologist experience, and patient spectrum. Fourth, emergency presentation, CT findings at emergency admission, and post-COVID incidence shifts were not consistently reported in the underlying DTA studies and therefore could not be synthesized quantitatively. Finally, the asymmetry in the Deeks’ funnel plot suggests the potential for publication bias or small-study effects, implying that the pooled estimates may reflect an optimistic summary of performance.

In summary, contemporary cross-sectional imaging, particularly dedicated MRE and CTE, demonstrates robust diagnostic accuracy for primary small-bowel tumors and is integral to clinical decision-making. Dedicated enterography techniques yielded the most favorable point estimates, but current evidence does not establish unequivocal superiority over conventional CT. Future research should prioritize prospective, multi-center studies with direct head-to-head comparisons of enterography protocols, standardized reporting of positivity criteria, clearer reporting of emergency presentations, and histology-specific analyses to refine guideline-level diagnostic sequencing strategies.

## Data Availability

The original contributions presented in the study are included in the article/**Supplementary Material**. Further inquiries can be directed to the corresponding author.

## Supplementary material

Supplementary Figure 1 Detailed SROC plots for specific subgroups. (A) SROC plots showing within-country heterogeneity for studies conducted in China. (B) SROC plots detailing the diagnostic accuracy of specific functional imaging tracers (^68^Ga-DOTATATE PET/CT vs. ^18^F-DOPA PET/CT).

Supplementary Figure 2 Overall diagnostic performance in the sensitivity analysis. Forest plots of the pooled sensitivity and specificity derived from the complete, non-deduplicated dataset (incorporating all 38 test arms). The squares represent individual test arm estimates with their 95% confidence intervals (CIs), and the diamonds represent the pooled estimates. This sensitivity analysis demonstrates the robustness and statistical stability of the primary findings.

Supplementary Figure 3 Subgroup analysis stratified by imaging modality (Sensitivity analysis). Forest plots comparing the diagnostic accuracy of dedicated enterography (MRE and CTE), conventional CT, and functional imaging modalities (e.g., ^18^F-FDG PET/CT, ^68^Ga-DOTATATE PET/CT) based on the complete dataset of 38 test arms.

Supplementary Figure 4 Subgroup analysis stratified by tumor type (Sensitivity analysis). Forest plots of diagnostic accuracy categorized by specific tumor histologies or classifications (e.g., generic small bowel lesions [SBL], neuroendocrine tumors [NET], gastrointestinal stromal tumors [GIST], and unknown/mixed types [UNK]) utilizing the complete dataset.

Supplementary Figure 5 Subgroup analysis stratified by the use of PET tracers (Sensitivity analysis). Forest plots evaluating the impact of PET tracers on diagnostic performance, comparing conventional anatomical/structural imaging modalities (No PET tracer) with functional imaging modalities (Yes PET tracer) across all 38 test arms.

Supplementary Figure 6 Subgroup analysis stratified by country of origin (Sensitivity analysis). Forest plots depicting the geographic distribution and corresponding diagnostic accuracy across different national cohorts (e.g., China, USA, Korea) based on the complete dataset.

Supplementary Table 1 PRISMA DTA Checklist.

Supplementary Table 2 Study-specific methodological quality assessment. Detailed tabular presentation of the QUADAS-2 scores for each included study, documenting judgments for risk of bias and applicability concerns across all four domains.
