# Supplementary figures and images for: Diagnostic accuracy of imaging modalities for primary small-bowel tumors: a systematic review and diagnostic test accuracy meta-analysis
Source: Front Oncol. 2026 Jun 26;16:1842330. doi: 10.3389/fonc.2026.1842330 (PMC13349763; doi:10.3389/fonc.2026.1842330)

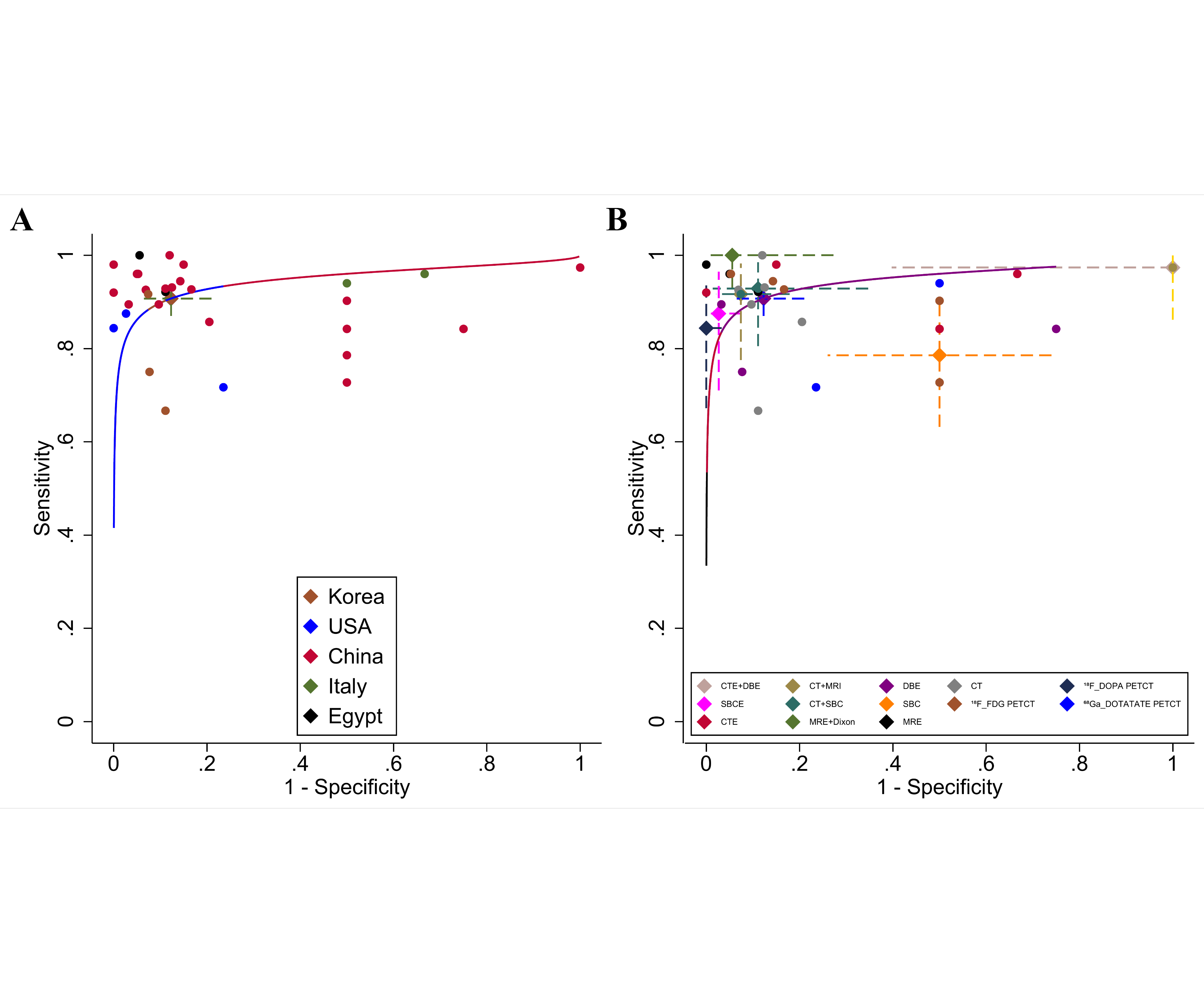

Supplement: Supplementary Figure 1 — Detailed SROC plots for specific subgroups. (A) SROC plots showing within-country heterogeneity for studies conducted in China. (B) SROC plots detailing the diagnostic accuracy of specific functional imaging tracers (68Ga-DOTATATE PET/CT vs. 18F-DOPA PET/CT). [file Image1.tiff]

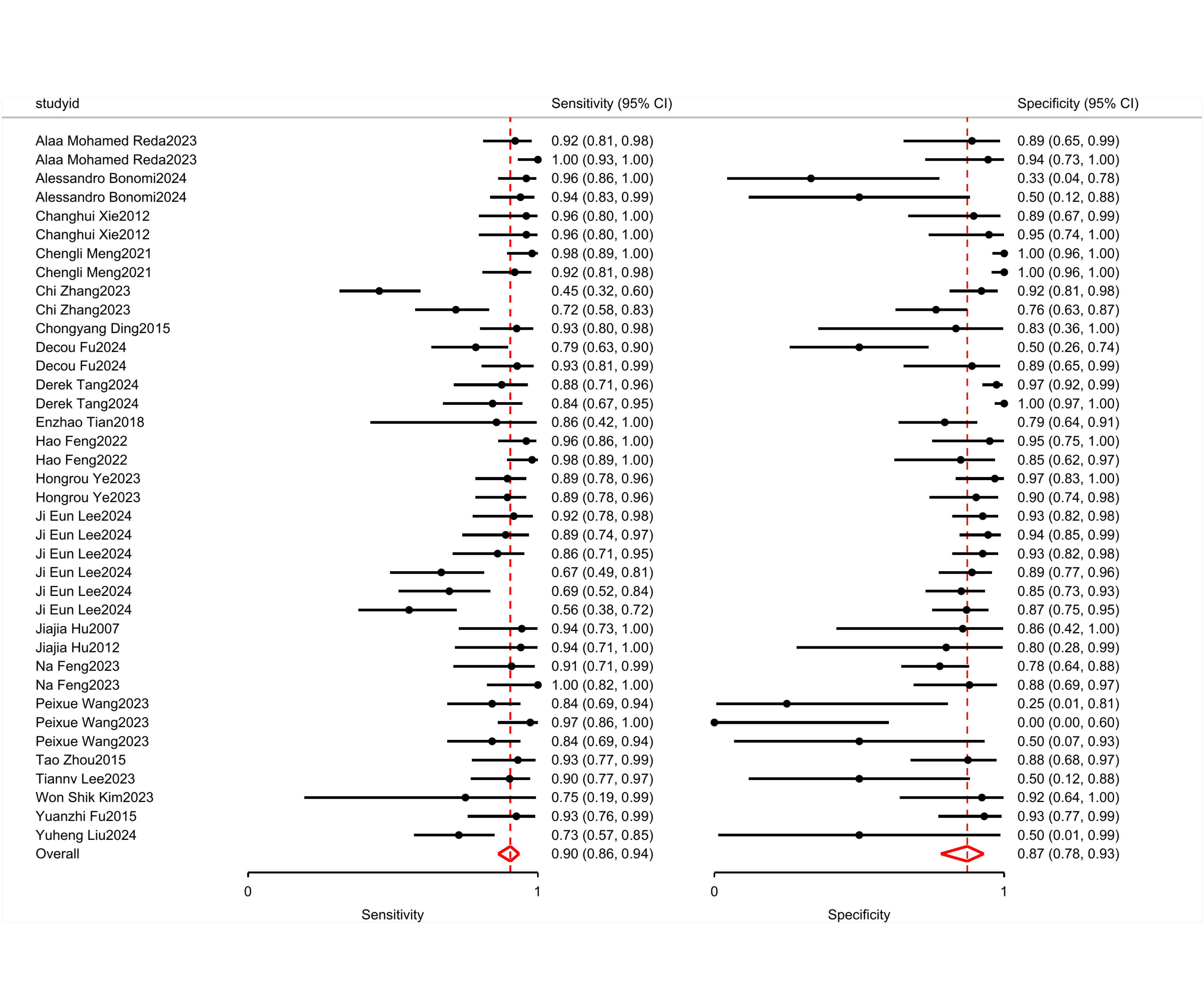

Supplement: Supplementary Figure 2 — Overall diagnostic performance in the sensitivity analysis. Forest plots of the pooled sensitivity and specificity derived from the complete, non-deduplicated dataset (incorporating all 38 test arms). The squares represent individual test arm estimates with their 95% confidence intervals (CIs), and the diamonds represent the pooled estimates. This sensitivity analysis demonstrates the robustness and statistical stability of the primary findings. [file Image2.tiff]

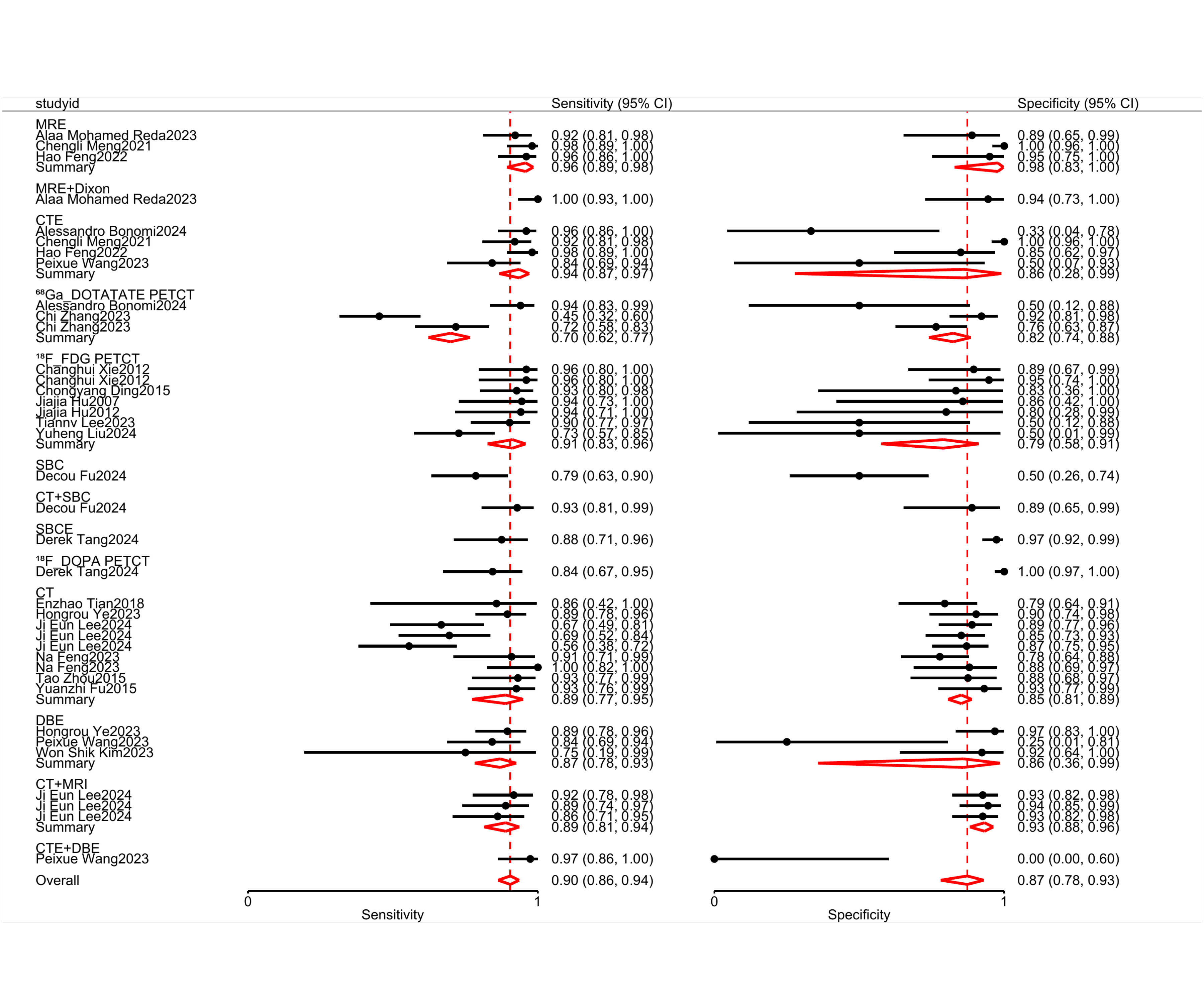

Supplement: Supplementary Figure 3 — Subgroup analysis stratified by imaging modality (Sensitivity analysis). Forest plots comparing the diagnostic accuracy of dedicated enterography (MRE and CTE), conventional CT, and functional imaging modalities (e.g., 18F-FDG PET/CT, 68Ga-DOTATATE PET/CT) based on the complete dataset of 38 test arms. [file Image3.tiff]

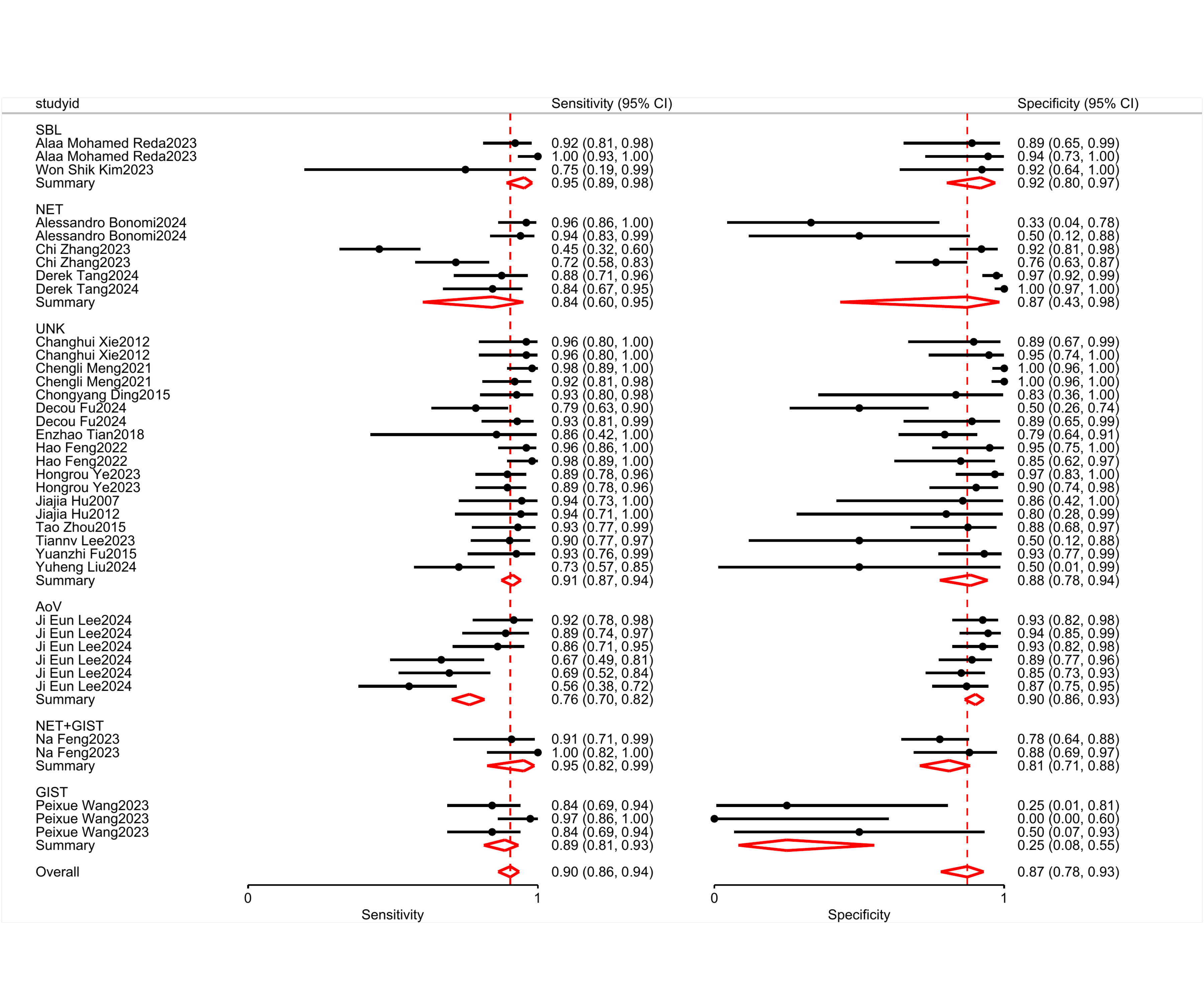

Supplement: Supplementary Figure 4 — Subgroup analysis stratified by tumor type (Sensitivity analysis). Forest plots of diagnostic accuracy categorized by specific tumor histologies or classifications (e.g., generic small bowel lesions [SBL], neuroendocrine tumors [NET], gastrointestinal stromal tumors [GIST], and unknown/mixed types [UNK]) utilizing the complete dataset. [file Image4.tiff]

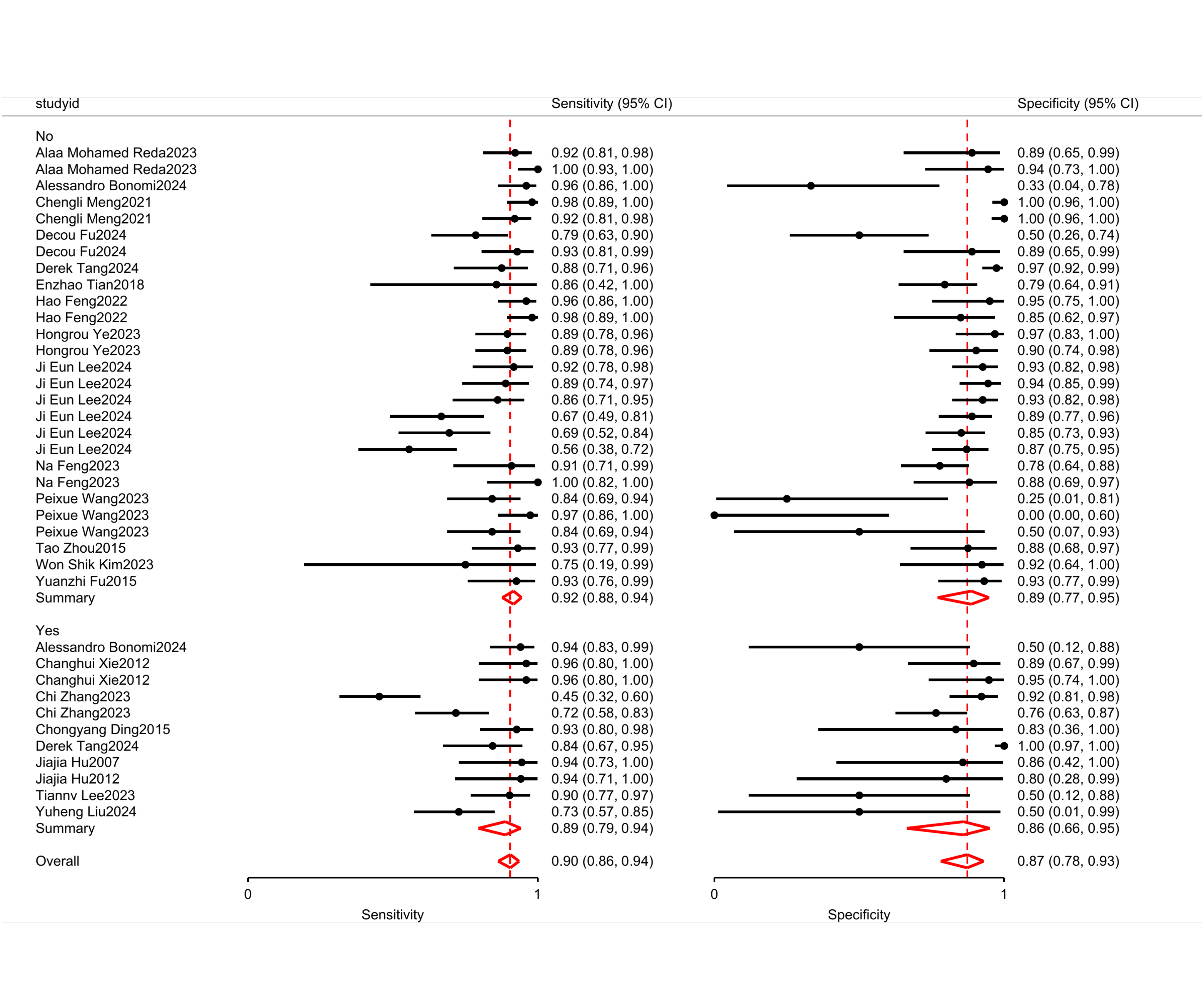

Supplement: Supplementary Figure 5 — Subgroup analysis stratified by the use of PET tracers (Sensitivity analysis). Forest plots evaluating the impact of PET tracers on diagnostic performance, comparing conventional anatomical/structural imaging modalities (No PET tracer) with functional imaging modalities (Yes PET tracer) across all 38 test arms. [file Image5.tiff]

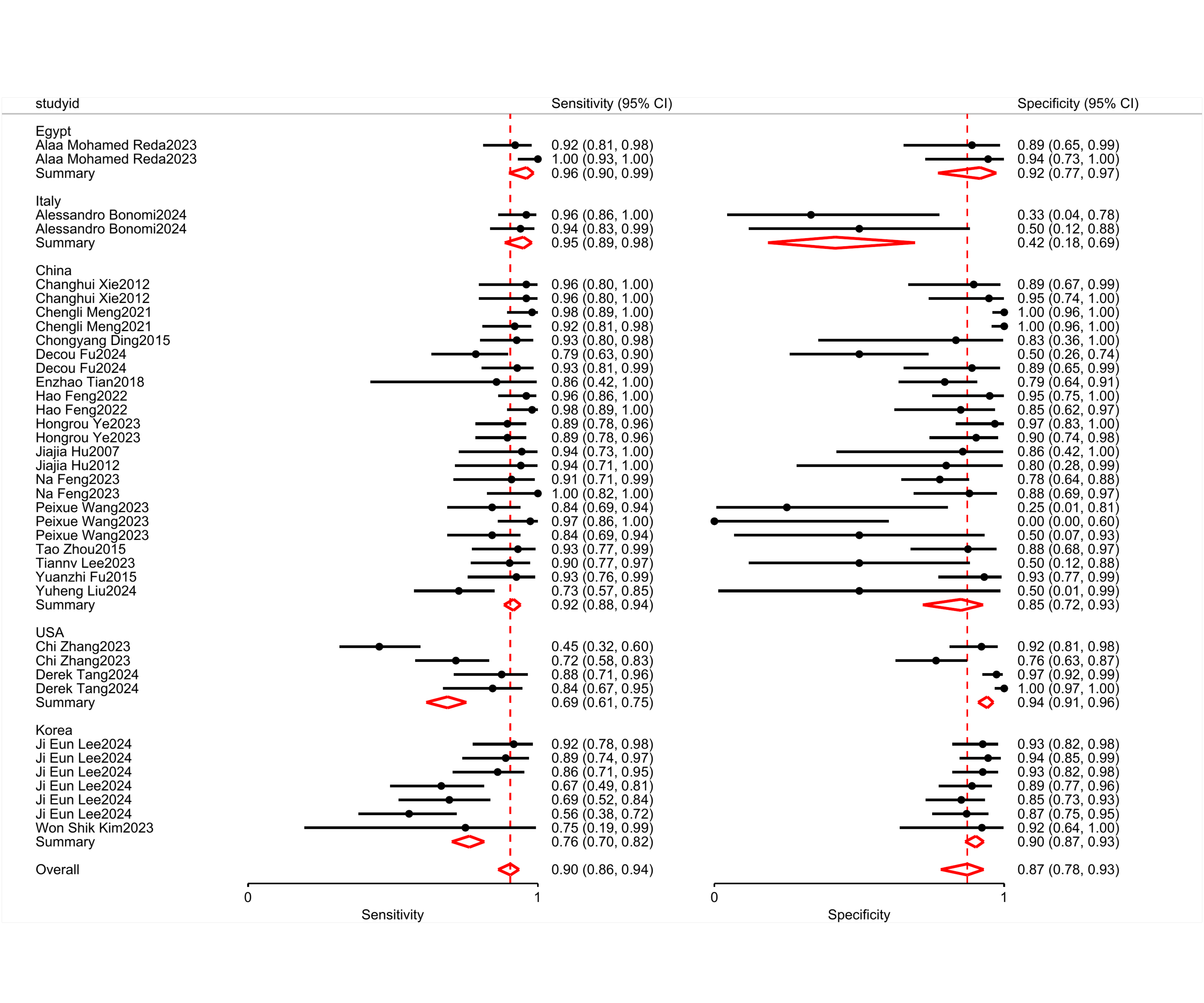

Supplement: Supplementary Figure 6 — Subgroup analysis stratified by country of origin (Sensitivity analysis). Forest plots depicting the geographic distribution and corresponding diagnostic accuracy across different national cohorts (e.g., China, USA, Korea) based on the complete dataset. [file Image6.tiff]
