# Supplementary material for: Diagnostic accuracy of imaging modalities for primary small-bowel tumors: a systematic review and diagnostic test accuracy meta-analysis
Source: Front Oncol. 2026 Jun 26;16:1842330. doi: 10.3389/fonc.2026.1842330 (PMC13349763; doi:10.3389/fonc.2026.1842330)
Supplement: Supplementary Table 2 — Study-specific methodological quality assessment. Detailed tabular presentation of the QUADAS-2 scores for each included study, documenting judgments for risk of bias and applicability concerns across all four domains. [file Table2.docx]

**Supplemental Table 2. Study-specific methodological quality assessment (QUADAS-2)**

| **Study (Year)** | **Domain 1: Patient Selection** | **Domain 2: Index Test** | **Domain 3: Reference Standard** | **Domain 4: Flow and Timing** | **Overall Risk of Bias** |
| --- | --- | --- | --- | --- | --- |
| Zhang et al. (2023) | Low | Some concerns | Low | Low | Some concerns |
| Wu & Chou (2024) | Some concerns | No information | High | No information | High |
| Wang et al. (2023) | Low | Low | Low | Low | Low |
| Tang et al. (2024) | Low | Low | Low | Low | Low |
| Shin et al. (2023) | Low | Low | Low | Low | Low |
| Reda et al. (2023) | Low | Some concerns | Low | Low | Some concerns |
| Lee et al. (2025) | Low | Low | Low | Low | Low |
| Kim et al. (2020) | No information | No information | No information | No information | High |
| Guo et al. (2023) | Low | Low | Low | Low | Low |
| Feng et al. (2023) | Low | Low | Low | Low | Low |
| Dong et al. (2024) | Low | Low | Low | Low | Low |
| Deepthi et al. (2025) | Low | Low | Low | Low | Low |
| Bonomi et al. (2024) | Low | Low | Low | Low | Low |
| Zhou et al. (2015) | Some concerns | No information | Low | No information | Some concerns |
| Ye et al. (2018) | Low | Low | Low | Low | Low |
| Changhui Xie-a (2012) | Low | Low | Low | Low | Low |
| Changhui Xie-b (2012) | Some concerns | Low | Low | Low | Low |
| Enzhao Tian (2018) | Low | Low | Low | Low | Low |
| Chengli Meng (2021) | Low | Low | Low | Low | Low |
| Yuheng Liu (2024) | Low | Low | Low | Low | Low |
| Tiannv Li (2014) | High | Low | Low | Low | High |
| Jiajia Hu (2012) | Some concerns | Low | Low | Low | Some concerns |
| Jiajia Hu (2007) | Some concerns | Low | Low | Low | Some concerns |
| Yuanzhi Fu (2015) | Some concerns | Low | Some concerns | Low | Some concerns |
| Decou Fu (2024) | Low | Low | Low | Low | Low |
| Hao Feng (2022) | Low | Low | Low | Low | Low |
| Chongyang Ding (2015) | Some concerns | Low | Low | Low | Some concerns |
